# Correction: Peguda et al. The Activity of Polyhomoarginine against *Acanthamoeba castellanii*. *Biology* 2022, *11*, 1726

**DOI:** 10.3390/biology12030470

**Published:** 2023-03-20

**Authors:** Hari Kumar Peguda, Rajamani Lakshminarayanan, Nicole A. Carnt, Zi Gu, Mark D. P. Willcox

**Affiliations:** 1 School of Optometry and Vision Science, University of New South Wales, Sydney 2052, Australia; 2 Ocular Infections and Anti-Microbials Research Group, Singapore Eye Research Institute, Singapore 169856, Singapore; 3 Ophthalmology and Visual Sciences Academic Clinical Program, Duke-NUS Graduate Medical School, Singapore 169857, Singapore; 4 Department of Pharmacy, National University of Singapore, Singapore 119077, Singapore; 5 School of Chemical Engineering, University of New South Wales, Sydney 2052, Australia

## Figure Legend

In the original publication [1] , there was a mistake in the legend for ** Figure 1—4 **. **Using a two-sample *t*-test and two-tailed distribution**. The correct legend appears below.

**Using one-way ANOVA**

**i. Figure 1: Change “(** *p* < 0.001 using a two-sample *t*-test and two-tailed distribution).” to “(** *p* < 0.001 using one way ANOVA).” 

**ii.** Figure 2: Change “(** *p* < 0.001, * *p* < 0.05 using a two-sample t-test and two-tailed distribution).” to “(** *p* < 0.001, * *p* < 0.05 using one way ANOVA).” 

**iii.** Figure 3: change “(** *p* < 0.001, * *p* < 0.05 using a two-sample t-test and two-tailed distribution).” to “(** *p* < 0.001, * *p* < 0.05 using one way ANOVA).” 

**iv.** Figure 4: change “(** *p* < 0.001, * *p* < 0.05 using a two-sample t-test and two-tailed distribution).” to “(** *p* < 0.001, * *p* < 0.05 using one way ANOVA).” **

## Text Correction

There was an error in the original publication. **Full name of PHMB missing in Introduction**. A correction has been made to **Introduction**, **Paragraph Number—3**:

** **Polyhexamethylene biguanide** **

**i.** Change “PHMB or chlorhexidine are widely used to treat this infection as monotherapies or in combination” to “Polyhexamethylene biguanide or chlorhexidine are widely used to treat this infection as monotherapies or in combination”.

The authors state that the scientific conclusions are unaffected. This correction was approved by the Academic Editor. The original publication has also been updated.
